# Update on the detection of frailty in older adults: a multicenter cohort machine learning-based study protocol

**DOI:** 10.18632/aging.206254

**Published:** 2025-05-21

**Authors:** Samuel Fernández-Carnero, Oliver Martínez-Pozas, Daniel Pecos-Martín, Armando Pardo-Gómez, Juan Nicolás Cuenca-Zaldívar, Eleuterio A. Sánchez-Romero

**Affiliations:** 1 Departamento de Fisioterapia, Universidad de Alcalá, Facultad de Enfermería y Fisioterapia, Grupo de Investigación en Fisioterapia y Dolor, Alcalá de Henares 28801, Spain; 2 Interdisciplinary Research Group on Musculoskeletal Disorders, Madrid 28014, Spain; 3 Physiotherapy and Orofacial Pain Working Group, Sociedad Española de Disfunción Craneomandibular y Dolor Orofacial (SEDCYDO), Madrid 28009, Spain; 4 Geriatrics Service, Puerta de Hierro Hospital, Majadahonda 28222, Spain; 5 Research Group in Nursing and Health Care, Puerta de Hierro Health Research Institute-Segovia de Arana (IDIPHISA), Majadahonda 28222, Spain; 6 Physical Therapy Unit, Primary Health Care Center “El Abajón”, Las Rozas de Madrid 28231, Spain; 7 Department of Rehabilitation Sciences, Florida Gulf Coast University, Fort Myers, FL 33965, USA

**Keywords:** frailty, ultrasound, sarcopenia

## Abstract

Background: This study aims to investigate the relationship between muscle activation variables assessed via ultrasound and the comprehensive assessment of geriatric patients, as well as to analyze ultrasound images to determine their correlation with morbimortality factors in frail patients.

Methods: The present cohort study will be conducted in 500 older adults diagnosed with frailty. A multicenter study will be conducted among the day care centers and nursing homes. This will be achieved through the evaluation of frail older adults via instrumental and functional tests, along with specific ultrasound images to study sarcopenia and nutrition, followed by a detailed analysis of the correlation between all collected variables.

Discussion: This study aims to investigate the correlation between ultrasound-assessed muscle activation variables and the overall health of geriatric patients. It addresses the limitations of previous research by including a large sample size of 500 patients and measuring various muscle parameters beyond thickness. Additionally, it aims to analyze ultrasound images to identify markers associated with higher risk of complications in frail patients. The study involves frail older adults undergoing functional tests and specific ultrasound examinations. A comprehensive analysis of functional, ultrasound, and nutritional variables will be conducted to understand their correlation with overall health and risk of complications in frail older patients.

Trial registration: The study was approved by the Research Ethics Committee of the Hospital Universitario Puerta de Hierro, Madrid, Spain (Act nº 18/2023). In addition, the study was registered with https://clinicaltrials.gov/ (NCT06218121).

## BACKGROUND

### The concept of frailty

Between 2015 and 2050, the global population aged 60 and above is projected to rise from 12% to 22% [1]. Advancing age correlates with a higher incidence of chronic ailments, frailty, dependency, and elevated healthcare expenditures [2–4]. Frailty is a complex medical condition marked by diminished strength, endurance, and physiological functions, heightening an individual's susceptibility to adverse outcomes, including mortality [5]. Its prevalence among the elderly ranges from 8% to 16% and is often intertwined with dementia [6] or cardiovascular disease [7]. Moreover, frailty in older adults frequently coexists with sarcopenia, an age-related syndrome characterized by progressive muscle mass and strength decline, heightening the risk of physical impairment and diminished quality of life [8].

### Pathophysiology of frailty and sarcopenia

Inflammaging presents a theory regarding frailty, suggesting that as individuals age, they experience a continuous low-level inflammation characterized by the release of proinflammatory cytokines from aging organs [5]. Individuals exhibiting frailty often display elevated levels of Interleukin-6 (IL-6), C-reactive protein (CRP), and Tumor Necrosis Factor-alpha (TNF-α), partially attributed to the prevalence of overweight/obesity in the elderly population. Moreover, frail individuals typically exhibit compromised immune function, diminished antibody production, or heightened mitochondrial oxidative stress, resulting in increased levels of inflammatory markers [5].

Individuals with sarcopenia commonly demonstrate heightened concentrations of biomarkers like CRP, Interleukin-1β (IL-1β), IL-6, or Interleukin-8 (IL-8), fostering a milieu of chronic inflammation. This condition is also associated with disruptions in amino acid metabolism and intestinal dysbiosis, leading to increased intestinal permeability to various microbes or their byproducts, subsequently triggering inflammatory responses and immune system dysregulation [9]. This sustained state of inflammation, coupled with hormonal shifts and other physiological processes, ultimately contributes to mitochondrial dysfunction within muscles, accelerated apoptosis of myonuclear cells, and impaired function of muscle satellite cells, culminating in muscular weakness [10].

### Diagnostic methods of frailty and sarcopenia

Various tools are employed for diagnosing frailty, including the “frailty phenotype,” which assesses weakness, slowness, exhaustion, low physical activity levels, and weight loss. Pre-frailty is recognized with the presence of one or two of these criteria, while frailty requires three or more [11, 12]. Other diagnostic approaches include the Frailty Index, Clinical Frailty Scale, or FRAIL scale [13].

Sarcopenia diagnosis hinges on low muscle strength, reduced muscle quantity/quality, and diminished physical abilities. Probable sarcopenia is diagnosed if the first criterion is met, sarcopenia if both the first and second are present, and severe sarcopenia if all three criteria are satisfied [14]. Additionally, assessment tools such as SARC-F questionnaires, muscle strength tests, diagnostic procedures like dual-energy X-ray absorptiometry (DXA), or physical assessments such as gait speed tests are employed [14].

DXA imaging allows quick determination of muscle tissue quantity, setting thresholds at <5.5 kg/m^2^ for women and <7.23 kg/m^2^ for men, with minimal radiation exposure and lower costs compared to computed tomography. However, DXA's efficacy is limited by low specificity and inability to assess muscle quality regarding fatty infiltration [15]. Computed tomography is the gold standard for evaluating muscle mass and quality but carries risks due to radiation exposure [15].

Ultrasound has emerged as a promising tool for diagnosing muscle conditions like frailty and sarcopenia, demonstrating comparable efficacy to DXA in assessing muscle mass and excellent reliability (ICC >0.9). Analysis of echo intensity in ultrasound images allows inference of muscle tissue quality [16]. The International Society of Physical Medicine and Rehabilitation has proposed an ultrasound-based algorithm for evaluating muscle mass, utilizing muscle thickness relative to body mass index, with loss of body mass defined as values <1 for women and <1.4 for men when comparing past and current measurements [16].

### Relevance and justification

Despite considerable research on frailty and sarcopenia in recent years, the prevalence of these is on the rise due, in part, to the growth of the elderly population and an increase in sedentary lifestyle among the elderly population. Current diagnostic methods such as DXA or Computed Tomography (CT) are expensive and involve radiation risk. Other methods, such as the FRAIL scales or the Clinical Frailty Scale, may require time and adequate patient cognition to perform. Faced with this dilemma, ultrasound has emerged as a method for evaluating muscle quality and quantity, being recommended by international organizations for use in the evaluation of frailty.

The correlation of the demographic variables, physical functionality tests and psychoemotional constructs that will be analyzed in this study with the ultrasound image obtained from the patients will improve the ultrasound diagnosis of frailty, providing new information that will facilitate the work of healthcare personnel in the diagnosis and management of frailty.

Similarly, the use of Machine Learning will allow institutions to extract data on different patient profiles, signs and symptoms of frailty and the different risk factors that affect frailty patients, which will improve treatments and favor the development of educational programs tailored to the patient's needs.

### Objectives

In this context, we designed the study to assess:

1. Update the diagnostic assessment of frailty by correlating several variables with the ultrasound image of the frail elderly patient.
2. We intend to collect and analyze data on functional capacity and quality of life variables on the evolution of musculoskeletal symptoms, as well as on pain and psychological variables. Similarly, it is intended to make a record of different profiles and subtypes of frail older adult patients to be stored with Machine Learning in order to establish therapeutic intervention plans that allow both the evaluation and treatment of patients.

## METHODS

### Study design

The present cohort study will be conducted in older adults diagnosed with frailty. A multicenter study will be conducted among the day care centers and nursing homes Residencia de Nuestra Señora de la Soledad y del Carmen (Colmenar Viejo, Madrid), Residencia San Camilo (Tres Cantos, Madrid), Centro de Día San Camilo (Tres Cantos, Madrid), Residencia San Luis de Gonzaga (Carretera de Colmenar Viejo), Hospital Príncipe de Asturias (Alcalá de Henares, Madrid) and Hospital Puerta de Hierro (Majadahonda, Madrid) from their diagnosis between April 2024 and June 2025, in the country of Spain. The study protocol was approved by the Research Ethics Committee of the Hospital Universitario Puerta de Hierro, Madrid, Spain (Act nº 18/2023). In addition, the study was registered with https://clinicaltrials.gov/ (NCT06218121), and will be conducted following the Strengthening the Reporting of Observational Studies in Epidemiology (STROBE) statement and checklist [17]. According to the Declaration of Helsinki, all patients will sign an informed consent form before inclusion and must agree that their clinical information will be published anonymously.

### Samples

A diagnosis of signs and symptoms of frailty by a geriatric physician from the research group (A.P.G.) and the centers' medical staff will be used as the main inclusion criterion. Frailty will be assessed and diagnosed using the frailty phenotype and the Clinical Frailty Scale.

As exclusion criteria:

- Acute myocardial infarction in the last 3 months and/or unstable angina pectoris.
- Uncontrolled arrhythmia, recent thromboembolism, and terminal illness.
- Patients with recent unloading of lower limbs (LL), or with UL/LL fractures in the last three months.
- Patients with a functional gait index of 1 (Inability to walk).
- Severe pain (7/10 Numeric Pain Rating Scale, NPRS).
- Previous neuromuscular pathology presenting with weakness.
- Medication that does not allow the patient's actual muscle reaction to be assessed.
- Severe cognitive impairment that would prevent collaboration and understanding of the tests to be performed.
- Cardiovascularly unstable patients and uncontrolled arterial hypertension.

For the cohort study, 500 frail older adult patients will be evaluated by means of instrumental and functional tests that assess their functional capacity, in addition to ultrasound imaging to study sarcopenia and nutrition, as well as psychological variables. The correlation between all functional, ultrasound, nutritional, and psychological variables will be analyzed. Through the Global Leadership Initiative on Malnutrition (GLIM) criteria diagnosis, anthropometric data (weight, height, and Body Mass Index-BMI) as well as analytical data including inflammation information (CRP and albumin) will be used to reach a diagnosis that allows comparison/correlation with the rest of the variable parameters [18]. The GLIM criteria provide a diagnostic system that has been accepted by the main international scientific societies in the field of clinical nutrition [19–21]. All available information will be collected during the follow-up in order to generate Machine Learning on the objective evolution and symptomatology of these patients, generating profiles that facilitate the most accurate and appropriate treatment for each patient.

### Materials

Measurement variables evaluated in patients include:

- Manual grip strength: The peak grip force was measured using a hydraulic hand dynamometer (JAMAR, Sammons Preston Rolyan, Sammons Court Bolingbrook, IL, USA) across a range of 0 to 90 kg. This device has been validated, shown to be reproducible, and deemed reliable for assessing grip strength in older individuals [22].
- Quality of life: measured with the EuroQol-5D, where mobility, personal care, daily activities, pain/discomfort, anxiety, or depression are assessed [23].
- Barthel Index: Assesses the level of independence of the subject with respect to the performance of activities of daily living [24].
- Trunk control test: Designed for the assessment of the patient's motor impairment [25].
- Intensity of Pain: Using the NPRS [26].
- Ultrasound image: The cross-sectional area and thickness of the rectus femoris muscle will be measured. In addition, contraction potential, contraction-relaxation velocity data will be taken by M-Mode ultrasound [27].
- The State-Trait Anxiety Inventory (STAI): Assessing state and trait anxiety [28].
- Kinesiophobia (TSK): Using the Tampa Kinesiophobia Scale, measuring fear of movement [29].
- Beck Depression Inventory (BDI): It measures depressive symptoms and the severity of depression [30].

**Figure 1:**
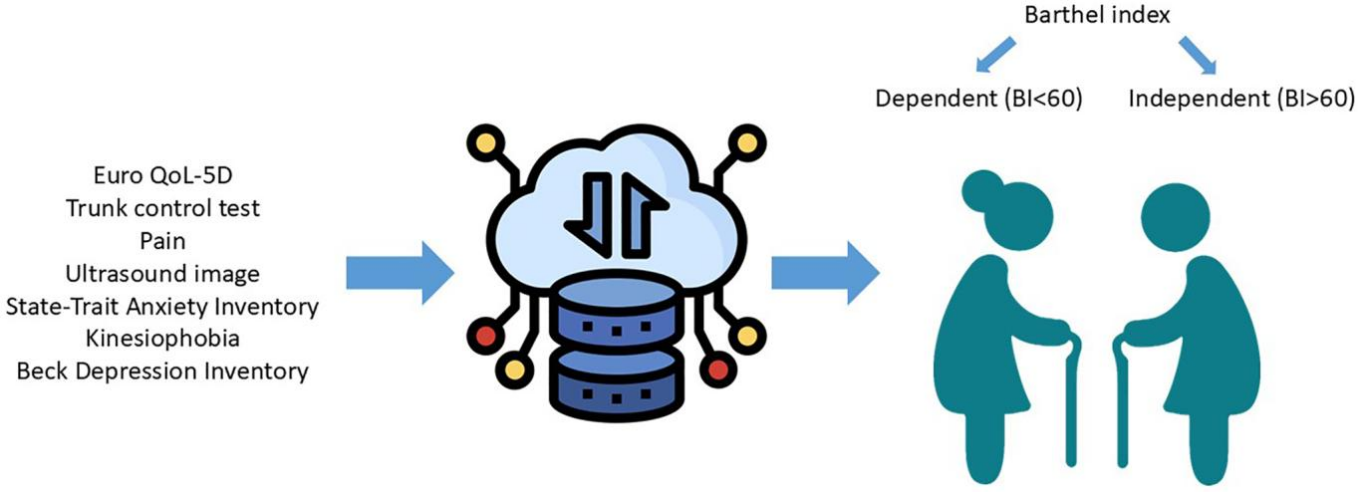
**Collection of variables in the older adult population of the study.** This visual summarizes the clinical, psychological, functional, and ultrasound-based variables collected in the frail elderly population.

**Figure 2:**
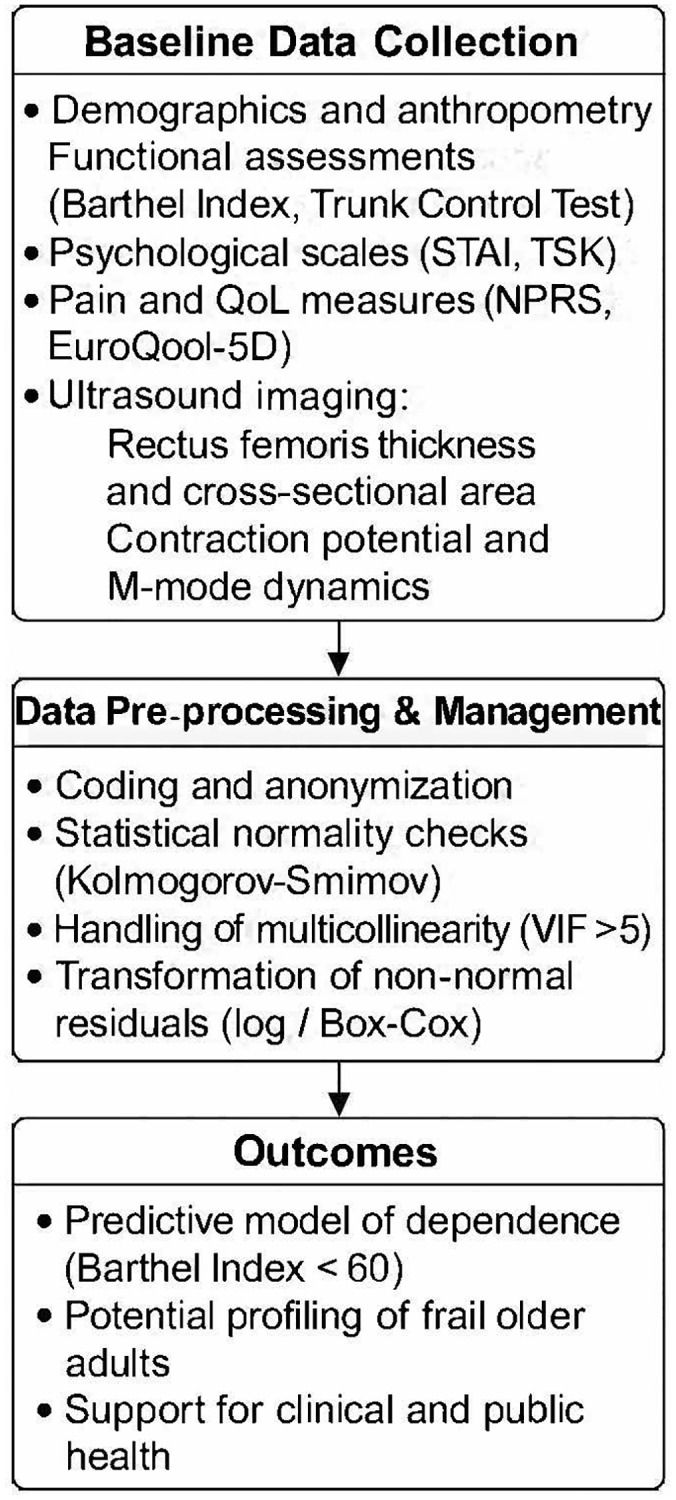
**Flowchart of the study protocol.** The diagram outlines the sequential phases of the multicenter cohort study, including participant recruitment, baseline assessments, data preprocessing, machine learning pipeline, and model outputs.

Figure 1 illustrates the collection of variables in the older adult population of the study. Figure 2 summarizes the study workflow, from participant recruitment and assessments to the machine learning pipeline for prediction and analysis.

### Statistical analysis

The R program version 4.3.1 will be used for statistical analysis. The level of significance will be set at *p* < 0.05. The distribution of quantitative variables, both baseline and outcome, will be tested with the Kolmogorov-Smirnov test with Lilliefors correction. Qualitative variables will be described in absolute values and frequencies and quantitative variables with mean and standard deviation.

Baseline and outcome variables differences using Barthel Index (BI) score dichotomized between dependent (BI < 60) and independent (BI > 60) patients [31] using *T*-test or U-Mann-Whitney for quantitative variables (depending on their distribution) and Fisher exact test for qualitative variables.

A multivariate analysis will be performed with a machine learning model through principal analysis using logistic regression, applying the model to 75% of the subjects and evaluating its accuracy in the remaining 25%, with the objective of creating a trained model that allows the development of therapeutic intervention plans that enable both the evaluation and treatment of patients. Model will be controlled by baseline variables with significant differences in univariate analysis.

Model will be constructed with dichotomized Barthel index as dependent variable and manual grip strength, EuroQoL-5D, NPRS, cross sectional area and thickness of the rectus femoris, STAI, TSK and BDI as explanatory variable. Logistic model will be selected instead of others used in machine learning (e.g. Random Forest, Support Vector Machines or Gradient Boosting) because it is computationally more efficient and faster and more easily interpretable.

We will follow these steps to perform model: a) delete variables with high multicollinearity that is, presenting a Variance Inflation Factor (VIF) highest than 5, b) check model assumptions, if residuals are not normality distributed, they will be transformed with log or Box-Cox transformation, if they present heteroskedasticity or residuals remain not normally distributed, robust bootstrap model will be applied, c) random split of data set into training (75%) and test (25%), c) train the model with cross validation (5-folders will be applied) and elastic net features selection (it combines lasso and ridge features selection), e) model performance will be assessed with confusion matrix, Receiver Operating Characteristic (ROC) curve and calculation of Area Under a Curve (AUC), sensitivity/specificity and F1 score and f) evaluation of variable importance.

### Image analysis

From each of the images the region of interest (ROI) will be manually selected from which the matrix of pixels from which the mean, standard deviation, variance, kurtosis and skewness, all first order parameters associated with the gray levels of each pixel, will be calculated.

The second-order parameters provide information on the spatial distribution of pixel gray levels from different methods:

Gray Level Co-Occurrence Matrices (GLCM), described by Haralick et al. [32, 33] which consist of comparing pairs of pixels separated by a certain distance (by default a value of 1 is used) and in an angular direction (0º, 45º, 90º and 135º) along the entire matrix, calculating the frequency with which certain gray levels appear in the image and their relationship between them. The parameters calculated are: homogeneity or second angular momentum, contrast, inverse difference, entropy, correlation, average sum, entropy sum, entropy difference, variance difference, variance sum, correlation measure A information, correlation measure B information, cluster shadow, cluster prominence, autocorrelation, dissimilarity, energy and maximum likelihood.

Gray-Level Run-Length Matrix (GLRLM) described by Galloway et al. [34] and calculated from the run-length statistic that represents a set of consecutive pixels having the same gray level in each of the four angular directions described along the whole matrix, obtaining the following parameters: gray level non-uniformity, high gray level run emphasis, long run emphasis, long run low gray level emphasis, run length non-uniformity, run percentage and short run emphasis.

Analysis of Local Binary Patterns (LBP) described by Ojala et al. [35] which consists of comparing the intensity of a central pixel, which is taken as a reference value, with those surrounding it, assigning them a value of 1 or 0 depending on whether their intensity is above or below the threshold value set by the central pixel, the resulting matrix is multiplied by the original, thus obtaining a matrix from which the final LBP parameter is calculated.

The blob analysis described by Nielsen et al. [36] is based on detecting areas close to each other with a similar eco-intensity called “blobs”. The number of blobs in each ROI is calculated as well as their average perimeter and standard deviation.

### Muscle architecture processing and analysis

Muscle thickness, pennation angle, and muscle fiber length will be assessed using ultrasound technology employing the SonoSite Edge II Ultrasound system (M Turbo Ultrasound system, SonoSite, Bothell, WA, USA), as described above [37]. Participants will be positioned in a seated posture with hip and knee angles set at 90°, ensuring that limb muscles are relaxed to prevent compression or deformation due to seating. To standardize measurements, the length of the femur (from the greater trochanter to the medial condyle) will be measured, and a mark will be placed at the lower third using a hypoallergenic pen for optimal probe placement.

A 15–6 MHz transducer probe (HFL 50×, SonoSite, USA), coated with water-soluble transmission gel (AquaSonic^©^) to enhance acoustic contact without causing pressure on the dermal surface, will be positioned perpendicular to the dermal surface of the vastus lateralis (VL) muscle, aligned with the muscle's median longitudinal plane. Three sagittal plane sonographs of the VL will be digitized, and the images will be transferred to a computer for analysis using ImageJ Software^©^ (National Institute of Health, Bethesda, MD, USA). To ensure consistency across the six participating centers, all ultrasound evaluations will be conducted using the same ultrasound system (SonoSite Edge II, SonoSite Inc., USA) equipped with the HFL 50× linear array transducer (15–6 MHz). Prior to data collection, all evaluators underwent a centralized training program, which included theoretical and practical sessions to ensure methodological homogeneity. Additionally, a pilot study was conducted to evaluate intra- and inter-rater reliability, with repeated measures analyzed using intra-class correlation coefficients (ICC). Excellent reproducibility was observed, with ICC values exceeding 0.90 for both muscle thickness and cross-sectional area.

All sonographic images will be obtained in a standardized position: participants seated with 90° hip and knee flexion, and the probe placed perpendicularly at the distal third of the thigh, as marked based on femur length. Water-based transmission gel will be used to avoid pressure artifacts. The acquisition protocol includes three sagittal plane images of the vastus lateralis, and a fourth M-mode image to assess contraction potential and relaxation velocity. All ultrasound operators were blinded to the frailty classification of the participants during image acquisition. The reproducibility of ultrasound measurements was confirmed during a pilot study prior to data collection, with intra- and inter-rater intraclass correlation coefficients (ICC) exceeding 0.90 for both muscle thickness and cross-sectional area, indicating excellent agreement.

### Cost-effectiveness evaluation

Given the research design, we will seek to compare the cost of using ultrasound vs. battery of tests, although we assume that the use of battery of tests will be more economical.

A secondary analysis will be performed that will mainly include the following elements of analysis to complete an economic evaluation of both alternatives:

1. Resource consumption and costs involved in both alternatives.
2. Using if possible, a social approach, differentiating it from the approach of the funder (Public Health Service).
3. Include a sensitivity analysis of the most influential variables on the results.

### Sample characterization

Sociodemographic information (such as age, gender, level of education, perceived financial status, and frequency of social activity participation) is gathered using self-administered questionnaires as predictors of physical activity and fall history. These questionnaires are detailed in this paper.

### Data collection procedure

Recruitment will begin by contacting different centers participating in this study to ask geriatric physicians for potential candidates. In addition, center staff will also distribute flyers to increase the chances of reaching all potential candidates. Candidates who wish to participate voluntarily should express their interest by contacting the research team by phone or through the associate geriatricians at the different centers. After the first contact, the eligibility criteria for including potential participants in the study will be verified, additional information will be provided if needed, doubts will be resolved and, finally, the informed consent for participation will be signed before proceeding with data collection. Before starting the measurement, all investigators involved in the measurement evaluation performed the same protocol evaluation to ensure that all investigators performed the same procedures when evaluating different outcomes. All researchers who participated in the measurement evaluation had demonstrated experience in qualitative research and had five or more years of clinical practice experience.

After enrollment in the study, patients will undergo an extensive evaluation of previous described outcomes. Validated and translated questionnaires will be used, and ultrasound evaluation will be performed by an experienced evaluator with more than 10 years of experience. Expected and unexpected serious adverse events during outcome measurement will be collected and reported to the research team. The study design schedule is shown in Table 1.

**Table 1:** Study design schedule.

**Timepoints**	**Enrollment**	**Assessments**
	**−1 week**	**0 week**
**Enrolment**		
Eligibility screen	X	X
Baseline	X	X
Informed consent	X	X
Medical history	X	X
Treatment history	X	X
Comorbidity	X	X
**Ultrasonography**	X	X
**Assessments**		
Manual grip strength	X	X
EQ-5D-5L	X	X
Barthel index	X	X
Trunk control test	X	X
BDI	X	X
VAS	X	X
GLIM	X	X
MRCSS	X	X
STAI	X	X
TSK	X	X
TUG	X	X
Adverse events		X

Timeline of procedures including enrollment, assessments, and ultrasound imaging. Abbreviations: EQ-5D-5L: EuroQol 5-dimensions 5-levels; BDI: Beck Depression Inventory; VAS: visual analogue scale; GLIM: Global Leadership Initiative on Malnutrition; MRCSS: Medical Research Council Sum Score; STAI: State-trait Anxiety Inventory; TSK: Tampa Scale of Kinesiophobia; TUG: Timed Up and Go test.

### Sample size calculation

The sample size was carried out based on the main objective and analysis of the study to evaluate which variables influence the level of dependence/independence through a multivariate logistic regression model, with Barthel index dichotomize as dependent variable, and manual grip strength, EuroQoL-5D, NPRS, cross sectional area and thickness of the rectus femoris, STAI, TSK and BDI as explanatory variable, for which a sample of 500 subjects was recommended according to Bujang et al. formula (100 + 50 × number of explanatory variables) and minimum sample size for logistic regression models [38–40].

### Data management

Every participant will receive an exclusive identification code, devoid of any personal details that could potentially reveal their identity. All data collected during the study will be securely stored in a locked cabinet at the local organizations, following a protocol aligned with the principles of Good Clinical Practice (GCP) guidelines. Upon completion of the study, the gathered data will be securely transferred to the research team's logistics department, where databases will be established. These databases will adhere to established protocols for archiving research materials, ensuring proper storage and accessibility.

### Dissemination policy

The study outcomes will be disseminated to the local organizations collaborating in the research, as well as to the professional sponsors focusing on promoting healthy aging. In addition, the findings will be made accessible to the general public through various platforms, such as official websites. The research team will also document the study results in numerous peer-reviewed publications for wider distribution and scrutiny.

### Data availability statement

Not applicable (this manuscript does not report data generation or analysis).

## DISCUSSION

The aim of this study is twofold: firstly, to investigate the correlation between muscle activation variables assessed via ultrasound and the comprehensive assessment of geriatric patients. This approach seeks to better understand how ultrasound findings may be related to the overall health and functional status of older patients. In addition, lastly published review found that ultrasound showed a low-to-moderate diagnostic test accuracy for sarcopenia, although included sample sizes was low (most studies less than 200 patients) and mostly only measured muscle thickness [41]. To counteract these limitations, this study has a large sample size with 500 patients and will measure muscle thickness but also cross-sectional area, as well as contraction potential and contraction-relaxation velocity with M-mode. Secondly, to analyze ultrasound images using histogram distribution to determine their correlation with mobi-mortality factors in frail patients. This approach will identify potential ultrasound markers that may be associated with a higher risk of complications in frail patients, as other studies found in the literature [42, 43].

To achieve these objectives, a study will be conducted on frail older adults, who will undergo a series of instrumental and functional tests designed to assess their functional capacity. Additionally, specific ultrasound images will be obtained to study sarcopenia and nutrition in this patient group. Subsequently, a comprehensive analysis of the correlation between all functional, ultrasound, and nutritional variables collected during the study will be conducted. This analysis will identify potential relationships between muscle activation, ultrasound image, and overall health and risk of complications in frail older patients.

### Implications and limitations

The potential implications of this study are substantial for both clinical practice and public health. The integration of ultrasound-derived muscle parameters with functional and psychological assessments, analyzed through machine learning, offers a scalable and cost-effective approach for the early detection of frailty. This may enable clinicians to stratify risk and tailor interventions in community-dwelling and institutionalized older adults, enhancing preventive care and reducing adverse outcomes associated with frailty.

Nonetheless, the study presents several limitations. First, the ultrasound measurements are operator-dependent, which introduces a risk of inter-rater variability. To mitigate this, we ensured strict standardization of procedures and training across all sites, along with reliability testing. Second, although the machine learning model will be internally validated using cross-validation and a separate test set, no external validation in a different population is planned in this protocol. Third, as participants are recruited by convenience sampling from institutional settings, there is a potential for selection bias and limited generalizability to broader older adult populations. Fourth, the accuracy of ultrasound imaging can be affected by the presence of subcutaneous fat, particularly in individuals with overweight or obesity. Excess adipose tissue may reduce image clarity, impairing precise delineation of muscle boundaries and affecting measurements of thickness and cross-sectional area. To address this limitation, we use a standardized acquisition protocol with high-frequency probes and minimal compression, and we plan to include anthropometric indicators such as body mass index (BMI) in the analysis to statistically adjust for this potential confounder. Lastly, while the current model focuses on predicting dependence based on the Barthel Index, other frailty dimensions (e.g., cognitive, social) may not be fully captured. These limitations will be addressed in future longitudinal studies with broader inclusion criteria and external validation.
